# Towards Enhancing the Robustness of Scale-Free IoT Networks by an Intelligent Rewiring Mechanism

**DOI:** 10.3390/s22072658

**Published:** 2022-03-30

**Authors:** Syed Minhal Abbas, Nadeem Javaid, Ahmad Taher Azar, Umar Qasim, Zahoor Ali Khan, Sheraz Aslam

**Affiliations:** 1 Department of Computer Science, COMSATS University Islamabad, Islamabad 44000, Pakistan; minhal.abbas514@gmail.com (S.M.A.); nadeemjavaidqau@gmail.com (N.J.); 2 School of Computer Science, University of Technology Sydney (UTS), Sydney, NSW 2007, Australia; 3 Automated Systems & Soft Computing Lab (ASSCL), Prince Sultan University, Riyadh 12435, Saudi Arabia; asscl@psu.edu.sa; 4 College of Computer and Information Sciences, Prince Sultan University, Riyadh 11586, Saudi Arabia; 5 Faculty of Computers and Artificial Intelligence, Benha University, Benha 13518, Egypt or; 6 Department of Computer Science, University of Engineering and Technology Lahore (New Campus), Lahore 54000, Pakistan; umar.qasim@uet.edu.pk; 7 Computer Information Science, Higher Colleges of Technology, Fujairah 4114, United Arab Emirates; zkhan1@hct.ac.ae; 8 Department of Electrical Engineering, Computer Engineering and Informatics, Cyprus University of Technology, Limassol 3036, Cyprus

**Keywords:** scale-free IoT networks, centrality measures, edge rewiring, malicious attacks, network optimization, random attacks, robustness

## Abstract

The enhancement of Robustness (R) has gained significant importance in Scale-Free Networks (SFNs) over the past few years. SFNs are resilient to Random Attacks (RAs). However, these networks are prone to Malicious Attacks (MAs). This study aims to construct a robust network against MAs. An Intelligent Rewiring (INTR) mechanism is proposed to optimize the network R against MAs. In this mechanism, edge rewiring is performed between the high and low degree nodes to make a robust network. The Closeness Centrality (CC) measure is utilized to determine the central nodes in the network. Based on the measure, MAs are performed on nodes to damage the network. Therefore, the connections of the neighboring nodes in the network are greatly affected by removing the central nodes. To analyze the network connectivity against the removal of nodes, the performance of CC is found to be more efficient in terms of computational time as compared to Betweenness Centrality (BC) and Eigenvector Centrality (EC). In addition, the Recalculated High Degree based Link Attacks (RHDLA) and the High Degree based Link Attacks (HDLA) are performed to affect the network connectivity. Using the local information of SFN, these attacks damage the vital portion of the network. The INTR outperforms Simulated Annealing (SA) and ROSE in terms of R by 17.8% and 10.7%, respectively. During the rewiring mechanism, the distribution of nodes’ degrees remains constant.

## 1. Introduction

Wireless Sensor Networks (WSNs) have a significant impact on increasing the capabilities of the Internet of Things (IoT) [1,2]. In WSNs, network devices are widely used for many practical applications, such as health [3], transportation [4,5,6], agriculture [7,8], and education [9]. However, the failure of these network devices causes a great loss in an entire network system. It affects the communication with the remaining network devices and damages the whole network [10]. The importance of protecting real-world networks from failures occurs in the following case. A power line of 1000 kV broke down on 25 January 2008 due to snowfall that affected the power grid network [11]. Moreover, the subprime mortgage crisis occurred in the same year on Wall Street, America which caused a great economic loss [12]. Therefore, maintaining the network functionality against these failures is still a challenging task in WSNs.

The network functionality is mostly affected by cyber attacks. They are mainly grouped into two categories: Random Attacks (RAs) and Malicious Attacks (MAs). RAs remove the nodes randomly from the networks and damage the network functionality, while MAs remove the important nodes having the maximum number of connections in the network. MAs are more effective as compared to RAs. Therefore, the network functionality is affected to a great amount by MAs as compared to the RAs. To resist these MAs, the network functionality is based on the network topology.

In the complex network theory, two main network topologies are considered: one is the Small World Networks (SWNs) and the other is the Scale-Free Networks (SFNs). SWNs have two properties, one is low average path length and the second is high clustering coefficient [13]. In SWNs, nodes contain different characteristics such as energy and communication range. These nodes are mostly used to make heterogeneous networks. In contrast, SFNs are used to construct homogeneous networks of nodes having similar characteristics [14]. The SFNs are independent of the network size [15]. Thus, even with the addition of nodes, the topological properties of SFNs remain constant. Therefore, most of the network topologies are constructed using the scale-free properties [15].

One property of SFNs is that they follow the power-law. The nodes having a high number of connections are very few in a network, while the majority of nodes have less connections. By this property, the SFNs are made resilient to the RAs [15]. However, they are vulnerable to MAs. This is because MAs remove important nodes having the maximum number of connections in the network. Therefore, a large number of connections are affected by the removal of the important node.

A measure is required to compute the network Robustness (R) against MAs. Schneider et al. [16] calculate the R of the network by performing MAs. The measure is used as an objective function to make the network robust [15]. It depends on the percolation theory by finding the behavior of the network after performing MAs [16]. These attacks remove nodes having the maximum connections in the network. The process is continued until the network is isolated into nodes. Therefore, the R measure is based on the Maximum Connected Subgraphs (MCS) by removing nodes having maximum connections.

With the significance of nodes, links provide connections between nodes and maintain network connectivity. Therefore, the removal of links affects the network connectivity directly. The measure that evaluates the network connectivity against the link attacks is considered [17]. During link attacks, the network connectivity is damaged by removing important links from the network. After removing these links, the R of the network is calculated against link attacks. The *R*-value depends on the connectivity of the network after link attacks. Hence, different types of link attacks can be considered for affecting network connectivity.

Multiple optimization algorithms are used to enhance the R of SFNs against MAs. These algorithms include Genetic Algorithm (GA) [18], Multiple Population Genetic Algorithm (MPGA) [19], Greedy Model (GM) [20], Differential Evolution (DE) [21], Natural Connectivity Model (NCM) [22], Elephant Herding Optimization (EHO) [23], Hill Climbing (HA) [15], Simulated Annealing (SA) [24], ROSE [25], etc. These algorithms provide the enhancement of network R against MAs. During the optimization process, the nodes’ degree distribution remains the same as the initial network.

The enhancement of the network R against MAs is still a challenging task. In [25], authors make an optimized SFN by performing random selection of edges and rewiring them. During the rewiring process, the R measure is used to calculate the network connectivity by removing nodes using the local information of the network. Moreover, the study is a continuation of work presented in [26]. The major contributions of this study are enlisted as follows:

- Closeness Centrality (CC) measure is utilized to determine the central nodes in the network using minimum computational time.
- An Intelligent Rewiring (INTR) mechanism is proposed to make an optimized SFN using the intelligent selection of nodes.
- Using the global information of nodes, the network is optimized against CC based node removal.
- The Recalculated High Degree based Link Attack (RHDLA) and the High Degree based Link Attack (HDLA) are proposed to affect the network connectivity effectively using the local information of nodes.

The article is organized by considering the following sections. The related work is discussed in Section 2. The INTR mechanism and its description are given in Section 3. The simulation results of the INTR mechanism are presented in Section 4, and the conclusions of the article are provided in Section 5.

## 2. Related Work

In a complex network, the resilience of the network is a major concern to maintain the network functionality. To evaluate the resilience of the network, Schneider et al. propose a measure R [16] that calculates the resilience of the network after removing nodes having the maximum number of connections. The resilience of the network depends on the connectivity of the remaining network after each node removal. Zeng and Liu et al. propose Betweenness Centrality (BC) measure by the removal of important links and calculate the network connectivity against them [17]. The measure evaluates the largest component of the network after removing links. Hence, based on the connected part of the network, the resilience of the network is calculated against link attacks.

In recent years, different heuristic algorithms are proposed for the enhancement of network R [15,18,24]. In [27], the authors propose a simple edge rewiring method to make a robust network. In this method, nodes’ degrees remain the same during the rewiring. However, the network R can be enhanced by the interconnection of similar degree nodes. In [15], the authors design a greedy algorithm to form a robust network. The algorithm enhances the *R*-values by random edge rewiring methods. In these methods, independent edges are selected to perform rewiring. If the *R*-value is increased, then the rewiring method is selected; otherwise, another pair of the independent edge is selected to perform the same method. The greedy algorithm improves the *R*-value of the network; however, due to randomness, the solution falls into the local optima. In [24], the authors enhance the *R*-value of the network to resolve the local optima problem with SA. In this algorithm, rewiring methods are performed with some advancement. In this algorithm, if the *R*-values decrease after the rewiring method, then the rewiring method is accepted with some probabilistic value.

The optimization algorithm is used in a single population for enhancing the *R*-value of the network in [18]. A local optimal result is obtained in a population that causes the premature convergence issue. To overcome the issue, the authors propose the method of co-evolution in multiple populations based on GA [19]. In the evolution based algorithm, crossover and mutation operations are used for the enhancement of network R. Using these operations in multiple populations causes computational complexity.

The connectivity of the SFN is affected due to the removal of central nodes. To determine the importance of central nodes, the authors considered a BC measure that selects a node by calculating the distance to all other nodes in the network [17,28]. The node has the minimum distance to all other nodes in the network. In addition, it passes the information between two nodes as a bridge and maximizes the communication in the network. However, the measure has high computational time to find the central node in the network. Therefore, an efficient measure is required to find the central node and fragment the network with minimum resources. Moreover, the network is optimized against the removal of nodes using the local information of the node [29,30].

In [25], authors utilize the angle sum and degree difference operations for enhancement of R in SFN. The random edge rewiring is performed based on these operations and such a network is constructed that resembles an onion-like structure. The structure shows the high *R*-value against MAs. However, the enhancement of network R is restricted due to random edge selection. In [29], a novel strategy is proposed to perform edge rewiring. These rewiring edges establish a robust network. In this network, similar degree nodes are connected and an onion-like structure is exhibited. However, the enhancement of network R is still a challenging task.

## 3. Initialization of Scale-Free Network and Robustness Measure

**Figure 1:**
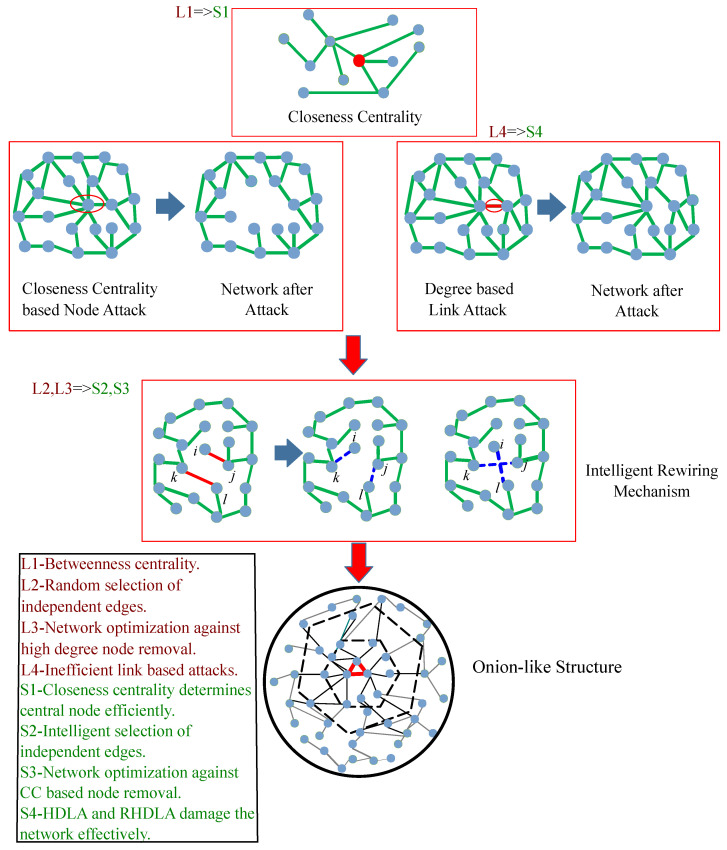
Proposed system model.

First, the initial SFN is constructed as shown in Figure 1. The CC measure is explained to find the central node. Then, the R of the network is calculated against the removal of the central node. Afterward, an optimized network is constructed based on the INTR mechanism. Finally, two link attacks are proposed to damage the network effectively.

### 3.1. Initial Scale-Free Network Construction

The Barabasi Albert (BA) model is utilized to construct an initial SFN. The model follows the power-law [14]. According to this law, nodes’ degrees are distributed in a certain way such that nodes with high degrees are few while those with low degrees are greater in number. The construction of the initial SFN is started from a clique. The clique is composed of a small number of nodes that are interconnected with each other and make an initial network. When a new node enters the network, the probability of each node is calculated using the nodes’ degrees. The new node connects to the node having high probability through the roulette wheel selection method. The process is continued and an SFN is established. However, the limited number of resources of a node makes it difficult to connect with multiple nodes in the network. Therefore, the network connections are restricted to a specific number in the network.

Both the communication range and degree of nodes are not considered establishing the SFN [15,24]. Therefore, the SFN is constructed by considering these constraints in the proposed work. In SFN, the nodes having a high number of connections are located at the center of the network, while other nodes having few connections are located at the boundary of the network by following the properties of power-law.

The construction of an initial SFN is discussed in Algorithm 1. After the random deployment of nodes, the $n_{i}$ node broadcasts the connection request message to all the nodes in its neighborhood (Line 4). The reply is sent by all the nodes to $n_{i}$ (Line 5). Ni represents nodes present in the range of node *i*. If these nodes have zero degrees, then an edge is made with the node that replies first (Line 7). In addition, the connection is made based on the probability if neighboring nodes $ne_{i}$ have different degrees (Lines 10). Moreover, the addition of nodes in the network is calculated using the edge density *m*. Therefore, the roulette wheel method is utilized to select *m* number of nodes (Lines 11). However, the degree of a node is restricted to a specific value because of limited resources of nodes. In the end, the neighboring list of nodes is updated (Line 15).   
**Algorithm 1:** Construction of an Initial SFN    **Input:** *A, N, m*    **Output: $list_{i}$**1: Procedure BA model (A)2:      *Random deployment of nodes*3:      **for all** $n_{i}\in N$ **do**4:         *$n_{i}$ broadcast the packet*5:         $n_{i}\leftarrow Neighbor\;Degree\left(\right)$6:         **If** Ni==0 **then**7:             *Make edge with the node that replies first*8:         **else**9:             **for all** $ne_{i}\in Ni$ **do**10:                *Calculate connection probability*11:                *Roulette wheel based node selection*12:             **end for**13:         **end if**14:      **end for**15: **end procedure**16: **Update: $list_{i}$**

### 3.2. Robustness Metric

The network connectivity is evaluated by various methods. In these methods, the removal of the node is performed from the network and *R* is calculated against them. Schneider et al. proposed a metric to compute the network *R* using the percolation theory [16]. In this theory, the network connectivity is analyzed against the MAs, and *R* of the network is calculated using Equation (1). MAs remove the nodes having the maximum connections in the network. During these attacks, the degrees of the nodes are updated. Using the updated degree values, MAs are performed on the highest degree nodes in each iteration. In this way, the connections of neighboring nodes are affected due to the removal process of high degree nodes. The process is continued until the whole network is damaged. The *R*-value of the network lies between 0 and 0.5. The minimum *R*-value is 0, which shows that the network is fully fragmented, whereas the maximum value of *R* is 0.5 for a fully connected network. However, the maximum *R*-value is less than 0.5 due to the constraints of nodes. The network *R* is computed against the MAs using the following equation:

(1) $$R=\frac{1}{N+1}\sum_{n=0}^{N-1}\frac{MCS_{n}}{N}.$$

In Equation (1), MCS represents the interconnected nodes in the largest component of network after removing *n* highest degree nodes. *N* represents the total number of nodes in the network. The consideration of each attack is represented by the summation operator, while the normalization factor is used to represent the different network sizes. The large value of *R* shows that the network is more robust against node attacks.

In the proposed model, the *R* measure is used against the node attack based on the CC by the motivation of [16]. The MAs based on CC are performed in the SFN. Using these attacks, the network connectivity is evaluated by removing nodes from the network.

### 3.3. Closeness Centrality Based Malicious Attacks

The connectivity of SFNs shows high *R* against RAs; however, these networks are vulnerable to MAs. These MAs are performed based on the significance of nodes in the network. In [25], authors utilize the degree of the node that gives the local information about nodes in the network. Using the local information, authors perform MAs and design a robust network against these attacks. In the proposed model, the CC measure is utilized to find the central node of the network as shown in Figure 1. It provides the shortest path from a node to every possible node in the network by calculating the distance of each pair of nodes [31]. The CC measure identifies nodes in the entire network based on the global information. The CC of a node *x* is determined in a network using Equation (2):

(2) $$c_{x}=1/\sum_{y}d\left(y,x\right).$$

In this equation, the centrality value of a node *x* is calculated by the reciprocal of distance summation from a node *x* to all nodes represented as *y* in the network. In this way, centrality values are computed for all the nodes in the network as given in Algorithm 2 and remove it. A node having the maximum value among them is selected (Line 4). The selected node has the minimum distance to all nodes in the entire network. Hence, the removal of the central node affects the connectivity by removing the connection of all neighboring nodes.   
**Algorithm 2:** Evaluate R Against Closeness Centrality Measure    **Input:** *G, N*    **Output:** *MCS, G_2_, R*1: **Procedure** Centrality Measure (G)2:      **for all** N∈G **do**3:         *Find CC of all nodes using Equation (2)*4:         *Select node having maximum value of CC in G*5:         *Remove the node and update G to G*_2_6:         *Calculate R using Equation (1)*7:      **end for**8: **end procedure**

In the system model, the connectivity of the network is damaged using the global information of nodes. These nodes point out the central node having the maximum CC value in the network. The connectivity of the network is evaluated after removing the central node. The removal process is continued until the network is split into nodes. Hence, the network R is calculated against the CC based node removal.

### 3.4. HDLA and RHDLA Based Link Attacks

In the SFN, nodes have a significant role to maintain the network connections. With the significance of nodes, links have their importance in network connectivity. These links communicate information between nodes in the network. The failure of important links affects the connectivity of a network [17]. Hence, the selection of important links is a challenging task.

In the proposed system model, HDLA and RHDLA are performed on the network as shown in Figure 1. These link attacks affect the network in different manners. In HDLA, the link is selected between the two highest degree nodes using the initial network information. It is because the importance of the link is based on adjacent nodes’ degrees. In HDLA, the node having the maximum connection in the network is selected first. Afterward, the connections with neighboring nodes are checked. According to the nature of SFNs, nodes having high degrees lie at the core of the network. Therefore, the link is selected that connects the two highest degree nodes and removes them from the network. The removal process is continued until the network is collapsed. In RHDLA, the degrees of nodes are updated after each removal of the link. Therefore, the removal of the link is based on the updated network information. Hence, both of these attacks affect the network connectivity by removing links that are closer to the center of the network.

The procedure of HDLA on the SFN is explained in Algorithm 3. First, the edge degree of the whole network using the adjacent nodes is calculated (Line 3). The edge degree of a link is calculated by the product of two adjacent nodes. Therefore, the information of all edge degrees is stored in $E_{d}$ (Line 3). The highest degree link is selected and removed from the network (Line 5). The network connectivity is calculated against the link removal (Line 6). The process is continued until the network is fragmented.    
**Algorithm 3:** HDLA and RHDLA    **Input:** *A, E, N*    **Output: MCS**1: **Procedure** High Degree Link Attacks (A)2:      **for all** E∈G **do**3:         *Calculate the edge’s degree of whole network as E_d_*4:         *Sort E_d_*5:         *Remove max(E_d_)*6:         *Calculate connectivity of the network*7:      **end for**8: **end procedure**

### 3.5. Optimization of Network Using Independent Edges

The optimization of SFN is based on the selection of independent edges. The two independent edges are selected if they fulfill the conditions that they are located within the communication range of nodes and have no additional edge among them. After the selection of two independent edges, the rewiring operations are performed between nodes of adjacent edges. Afterward, the network *R* is calculated against the MAs. If the first rewiring increases the *R*-value of the network, then the network is updated. If the *R*-value is decreased due to the first rewiring, then the alternative rewiring operation is performed. The optimization of network *R* is done only if the *R*-value is increased. If both rewirings are failed to increase the *R*-value of the network, then the initial *R*-value of SFN is considered.

### 3.6. INTR Mechanism

The INTR mechanism is proposed to optimize the network *R* against the MAs. In this mechanism, rewiring operations are performed to form an onion-like structure. The structure shows a high *R*-value against the MAs in the network. The nodes having high degrees are located at the center of the network. The degree of nodes decreases hierarchically from the center of the network towards the boundary. By analyzing the center of the network, it is found that there are strong connections between high degree nodes that make the network robust against MAs.

The INTR mechanism is used to make the network robust against the CC based MAs. MAs utilize the global information of nodes and remove the central node. Based on the MAs, the network optimization is performed by rewiring the connections between high and low degree nodes and making a robust network. In this mechanism, the intelligent selection of independent edges and rewiring operations makes an onion-like structure.

Initially, we consider the two nodes having the highest degrees in the entire network. After the selection of the highest degree nodes, the neighbors of these nodes are calculated using the local information. From the neighbor’s set, the two lowest degree nodes are selected. In this way, two edges are marked using the information of neighboring nodes. These edges are independent if they have no adjacent nodes other than these nodes and lie in the same communication range. If the independent edges fulfill the independence condition, then the rewiring process is performed. After the first rewiring process, the *R*-value of the network is calculated against the CC based node removal. If the *R*-value is enhanced, then the rewiring process is considered. Otherwise, another rewiring process is performed between the selected nodes. The process is continued until the *R*-value is increased. Hence, the INTR mechanism optimizes the network *R* using the intelligent rewiring that makes the onion-like structure.

In Algorithm 4, the highest degree node *i* is selected in the network (Line 3). The lowest degree node in the neighbor of the highest degree node is calculated and marked *j* (Line 4). The same steps are performed for the second-highest degree node as *k* and its lowest degree neighboring node *l* (Lines 5 and 6). Then, the independence of edges is checked (Line 8). If the edges are independent, then the rewiring process is performed. Afterward, the *R* of the network is calculated, and the graph and adjacency matrices are updated (Lines 10 and 11). Using the INTR mechanism, the network exhibits an onion-like structure that is resilient to MAs.   
**Algorithm 4:** INTR Mechanism    **Input:** *N, E, G*    **Output:** *R, A*1: **Procedure** INTR Based Edge Rewire (A)2:      **for all** N∈G **do**3:         *Find the highest degree node and mark it as i*4:         *Find the lowest degree neighbor node j based on i*5:         *Find the second highest degree node and marked k*6:         *Find the lowest degree neighbor node l based on k*7:         *Mark edges ij and kl*8:         **if** *both edges are independent from E*9:             *Rewire edge (i,k) and edge (j,l)*10:             *Calculate R*11:             *Update A and G*12:         **end if**13:      **end for**14: **end procedure**

## 4. Simulation Results and Discussion

**Figure 2:**
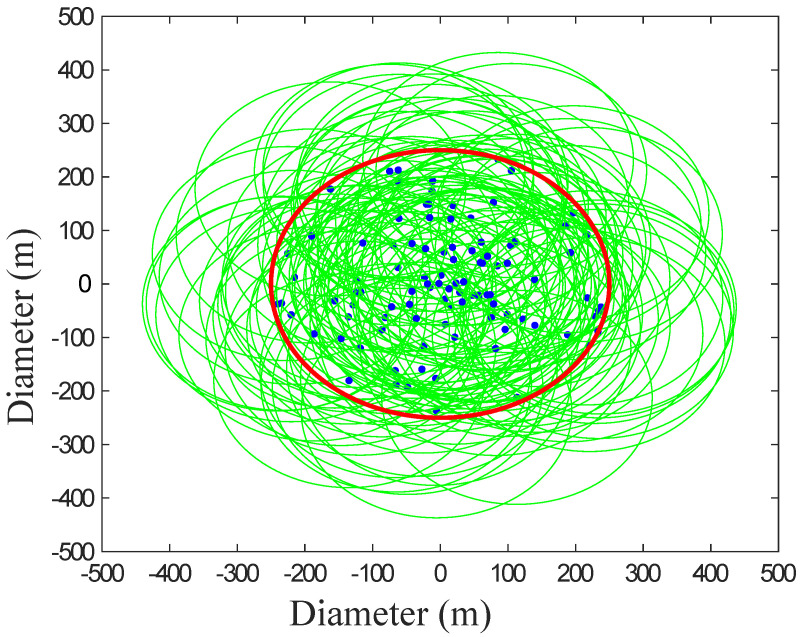
Deployment of nodes.

The evaluation of R in the SFN is discussed here. The nodes are randomly deployed in an area of 500 × 500 m^2^ as shown in Figure 2. The edge density *m* of the network is considered as 2. The maximum nodes’ degrees is restricted to 25 by the constraints of nodes while the communication range of each node is 200 m as shown in Figure 2. Table 1 shows solutions for the identified limitations, along with validations. All the simulations are averaged over 10 independent iterations.

### 4.1. Evaluation of Centrality Measures with Computational Time

**Figure 3:**
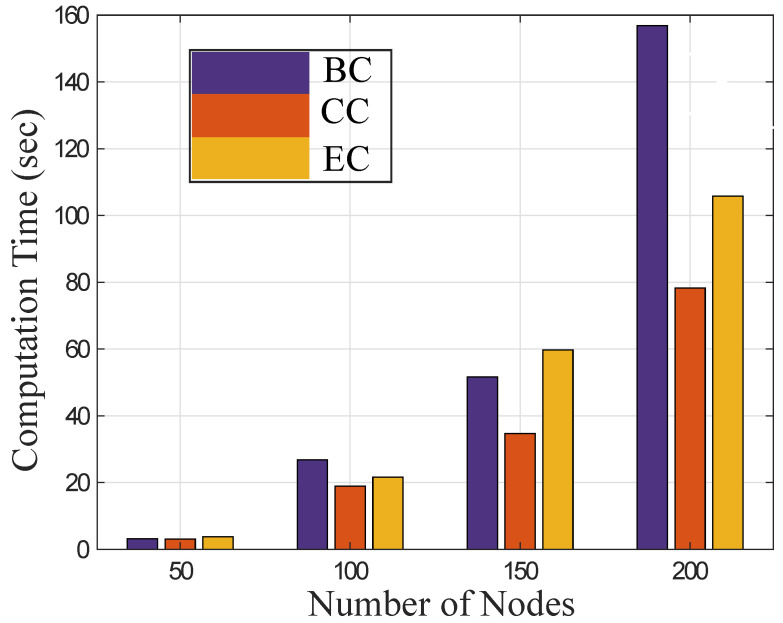
Evaluate computational time with different centrality measures.

The computational time of different centrality measures in the SFN is shown in Figure 3. The centrality measures find the central nodes with different criteria in the network. By increasing the sizes of the network, centrality measures BC, CC, and EC determine the central nodes and remove them from the network. Therefore, the connectivity of the network is affected by different centrality based MAs.

The performance of CC outperforms BC and EC with different numbers of nodes in Figure 3. It calculates the distance of each pair of nodes and selects the central node having a minimum distance with all other nodes in the network. The central node is situated close to all nodes in the entire network. Therefore, CC takes less computational time to find central nodes and remove them from the network, while BC and EC incur high computational time to damage the network by removing central nodes. This is because BC finds the central node based on a node that acts as the bridge between two other nodes in the network, while EC works with the recursive operations to find central nodes that incur large computational time.

### 4.2. Network Connectivity against Link Attacks

**Figure 4:**
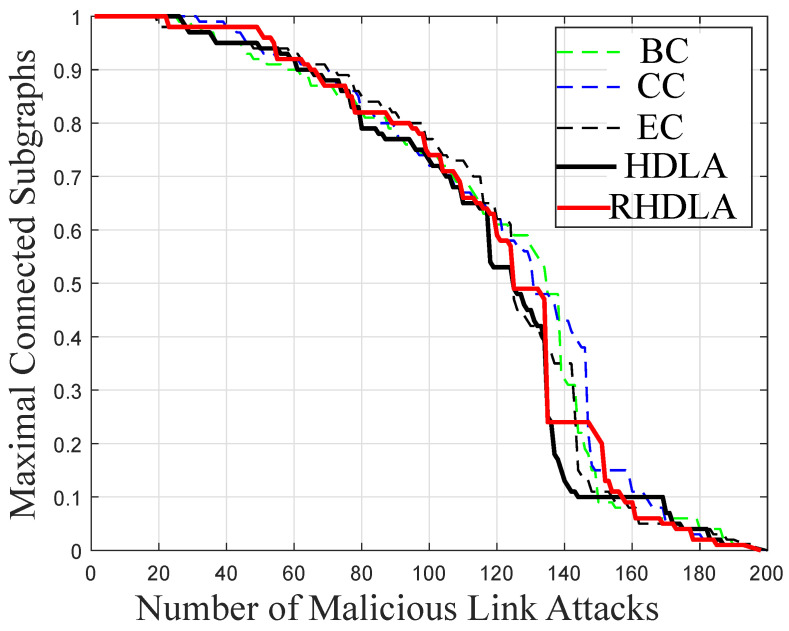
Centrality based link attacks.

Figure 4 represents the connectivity of the network affected by the removal of links. Different centrality measures BC, CC, and EC remove the important links and damage the whole network. The effectiveness of RHDLA and HDLA is found better than CC, BC, and EC in Figure 4. HDLA removes links in a sequential order from a high to low degree using the initial network information, while RHDLA removes the link and recalculates the nodes’ degrees of the entire network. Using the updated degree information, the rest of the links are removed from the network.

The network connectivity is affected most at the 80th HDLA compared with BC, CC, and EC based link attacks. With the increasing number of link attacks, HDLA consistently performs better than other centrality based link attacks. The reason is that HDLA removes the links in a descending order based on the initial network information of nodes’ degrees.

Furthermore, Figure 4 shows that RHDLA and HDLA affect the network connections with limited resources using the local information of the nodes, as compared to BC, CC, and EC that use global information. Since the SFN is a dense network, a large number of links are interconnected with each other. The removal of links in a dense network takes high computational resources. Therefore, HDLA and RHDLA perform effectively as compared to CC, BC, and EC based link attacks.

### 4.3. Robustness against Random and Malicious Attacks

**Figure 5:**
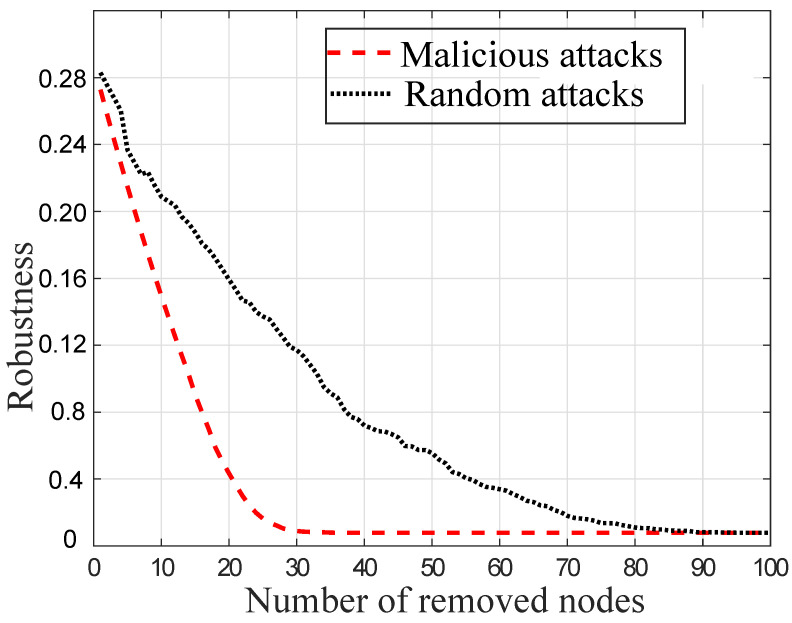
Random and malicious attacks.

SFNs are highly robust to RAs and are weak against MAs. Figure 5 shows the network *R* by the removal of nodes using these attacks. The optimized network has a 0.28 *R*-value. By increasing the number of attacks, the *R*-value of the optimized network is decreased as shown in the figure. It shows that the number of nodes that are removed against the MAs is greater as compared to the RAs. This is because MAs remove the maximum degree of nodes. In the figure, the MAs fragment the whole network at the 27th attack, while RAs damage the network connectivity at the 80th attack. It shows that the connectivity of the network is greatly affected by MAs as compared to RAs.

In Figure 5, the connectivity of SFN is less affected by the RAs. In RAs, there is a high probability to affect the low degree nodes in the network. This is because most of the nodes have low degrees in SFN. Therefore, RAs remove random nodes from the network and damage the network, while MAs select the nodes having high degrees and remove them. The links that are associated with these nodes are also removed. Therefore, a large number of connections are removed by the MAs.

### 4.4. Centrality Based Node Attacks

**Figure 6:**
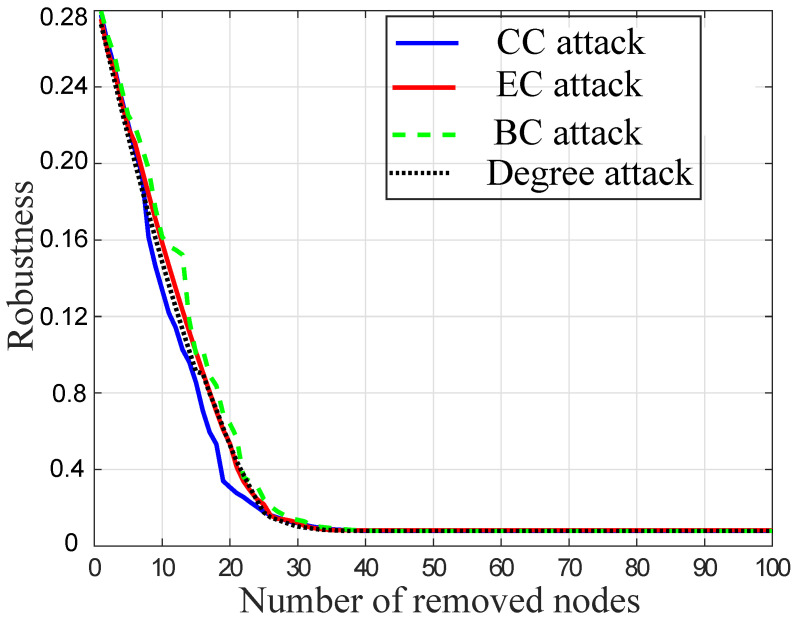
Centrality based node attacks.

The network R is evaluated against different types of centrality based node attacks in Figure 6. These attacks include CC, EC, BC, and degree attacks. In the figure, the optimized network has the 0.28 *R*-value. The connectivity of the network is analyzed after performing different centrality based node attacks. The *R*-value of the optimized network is quickly decreased against these centrality based attacks.

In the figure, the removal of nodes is analyzed against CC, EC, and BC attacks. The performance of CC is effective as compared to BC and EC in terms of computational cost. It is because the performance of BC and EC attacks is reduced when the network is dense. As the SFN is a dense network, both BC and EC therefore consume high computational resources by collecting global information from all nodes. Therefore, CC incurs less computational time to collect the global information and damage the entire network. Moreover, the CC attack is more effective as compared to the degree attack. This is because CC requires the global information of nodes to find the central node while the degree utilizes the local information of nodes. Therefore, CC damages the network effectively by removing the central nodes of the network compared with the degree based attack.

### 4.5. Evaluate the Power-Law Distribution

**Figure 7:**
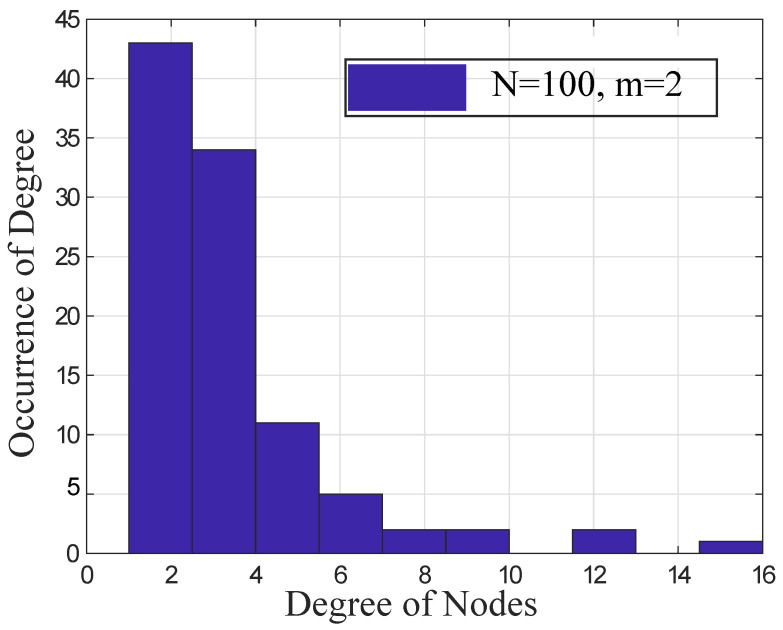
Power-law distribution.

The power-law property of SFNs is evaluated using the distribution of nodes’ degrees in the optimized network as shown in Figure 7. According to the definition of the power-law, a very small number of nodes having a high degree and a high number of nodes having a low degree exist in the network. If the distribution of nodes’ degrees is changed, then the property of SFNs is not followed in the optimized network. Therefore, it is important to check that the distribution of nodes’ degrees is following the power-law.

In Figure 7, *N* = 100 and *m* = 2. The *y*-axis shows the occurrence of nodes’ degrees in the entire network. The *x*-axis shows the degree of nodes in the optimized network. The figure shows that the occurrence of nodes’ degrees is gradually decreased by increasing the nodes’ degrees. It shows that the nodes having two degrees are mostly found in the network as compared to nodes having high degrees by considering power-law degree distribution. Therefore, the INTR mechanism makes a robust network that follows the property of SFN.

### 4.6. Comparison with Existing Algorithms

**Figure 8:**
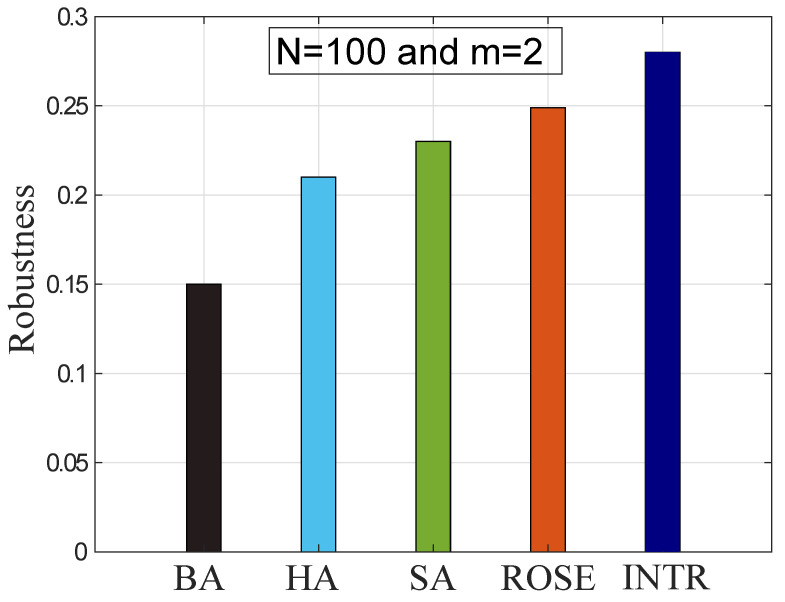
Evaluation of robustness with existing algorithms.

In Figure 8, the network R is evaluated using the INTR mechanism and is compared with SA and ROSE algorithms in the same environment. The performance of the proposed mechanism is enhanced by 17.8% and 10.7% as compared to SA and ROSE, respectively. This is because INTR rewires the edges between high and low degree nodes that enhance the *R*-value of the network. The rewiring process makes the connections of similar degree nodes and exhibits an onion-like structure. The structure shows high resilience against MAs. In this structure, when the MAs are performed on the central node, then the neighboring nodes replace its functionality with the removal node and maintain the network connections. In addition, the distribution of nodes’ degrees is unchanged during the entire rewiring process. The performance of SA and ROSE is less effective to enhance the network *R* due to the random selection of connections and their rewiring.

## 5. Conclusions

The BA model is utilized to construct the SFN by the consideration of the limited degree and the communication range of nodes. The INTR mechanism is proposed to optimize the *R* in the SFN that shows high resilience to MAs. In this mechanism, edge rewiring is performed between high and low degree nodes to enhance the network *R* against MAs. Based on the global information of nodes, CC finds the central nodes to damage the entire network using less computational resources as compared to BC and EC. Therefore, the optimization is performed by removing the node based on CC and rewiring the edges to enhance the network *R*. In addition, HDLA and RHDLA are performed to damage the network connectivity. HDLA removes the important links based on the initial network information, while RHDLA depends on the updated network information. Both these link attacks damage the network connections using less computational resources. Through simulations, INTR enhances the network *R* effectively by rewiring edges in an intelligent manner. The enhancement of network *R* from SA and ROSE is about 17.8% and 10.7%, respectively. In addition, the distribution of nodes’ degrees remains unchanged throughout the optimization process. Hence, the connectivity of nodes in an INTR based network exhibits an onion-like structure that shows high resilience to MAs. The INTR mechanism is restricted to homogeneous networks in which nodes have similar properties. Moreover, the network is optimized against a specific type of attack. In the future, we will consider heterogeneous networks and enhance the network *R* against various types of attacks. In addition, we will consider real-world networks including transportation networks, power grid networks, and airline networks to analyze the connectivity against attacks.

## Figures and Tables

**Table 1:** Mapping table.

Limitations	Solutions	Validations
**L1:** The BC measure determines the central node in the network inefficiently [17].	**S1:** The CC measure determines the network’s central node in less computational time.	**V1:** The computational time of different centrality measures is evaluated in Figure 3.
**L2:** No specific criteria for selection of independent edges [24].	**S2:** INTR is proposed to select the independent edges between high and low degree nodes for optimizing the network R.	**V2:** The R value evaluates the overall performance of network in Figure 8.
**L3:** Network is optimized only against high degree node removal [21,25,32].	**S3:** Network is optimized against high CC based node removal.	**V3:** The network performance is evaluated with R values in Figures 5, 7 and 8.
**L4:** The network connectivity is affected by link attacks using high computational resources [17].	**S4:** Two attacks HDLA and RHDLA are introduced that damage the network effectively.	**V4:** The network connectivity is validated by performing different centrality based link attacks in Figure 4.

## Data Availability

Not applicable.

## Abbreviations

The following abbreviations are used in this manuscript:

**Abbreviations and Acronyms**	**Description**
BA	Barabasi Albert
BC	Betweenness Centrality
CC	Closeness Centrality
DE	Differential Evolution
EHO	Elephant Herding Optimization
GA	Genetic Algorithm
GM	Greedy Model
HA	Hill Climbing
HDLA	High Degree based Link Attacks
INTR	Intelligent Rewiring
IoT	Internet of Things
MAs	Malicious Attacks
MCS	Maximum Connected Subgraphs
MPGA	Multiple Population Genetic Algorithm
NCM	Natural Connectivity Model
RAs	Random Attacks
R	Robustness
RHDLA	Recalculated High Degree based Link Attacks
SA	Simulated Annealing
SFNs	Scale-Free Networks
SWNs	Small World Networks
WSNs	Wireless Sensor Networks
*A*	Adjacency matrix of network
$c_{x}$	Closeness of a node *x*
d(y,x)	Shortest distance between *y* and *x*
*E*	Number of edges in graph
*G*	Current graph
$G_{2}$	Updated graph
*m*	Edge density
*N*	Total number of nodes
